# Boron-doped diamond semiconductor electrodes: Efficient photoelectrochemical CO_2_ reduction through surface modification

**DOI:** 10.1038/srep38010

**Published:** 2016-11-28

**Authors:** Nitish Roy, Yuiri Hirano, Haruo Kuriyama, Pitchaimuthu Sudhagar, Norihiro Suzuki, Ken-ichi Katsumata, Kazuya Nakata, Takeshi Kondo, Makoto Yuasa, Izumi Serizawa, Tomoaki Takayama, Akihiko Kudo, Akira Fujishima, Chiaki Terashima

**Affiliations:** 1 Photocatalysis International Research Center, Tokyo University of Science, 2641 Yamazaki, Noda, Chiba 278-8510, Japan; 2 Faculty of Science and Technology, Tokyo University of Science, 2641 Yamazaki, Noda, Chiba 278-8510, Japan; 3 ORC Manufacturing Co., Ltd, 4896 Tamagawa, Chino, Nagano 391-0011, Japan; 4 Environmental and Sustainability Institute, University of Exeter, Penryn, Cornwall TR10 9EZ, UK; 5 Faculty of Science, Tokyo University of Science, 1-3 Kagurazaka, Shinjuku, Tokyo 162-8601, Japan

## Abstract

Competitive hydrogen evolution and multiple proton-coupled electron transfer reactions limit photoelectrochemical CO_2_ reduction in aqueous electrolyte. Here, oxygen-terminated lightly boron-doped diamond (BDD_L_) thin films were synthesized as a semiconductor electron source to accelerate CO_2_ reduction. However, BDD_L_ alone could not stabilize the intermediates of CO_2_ reduction, yielding a negligible amount of reduction products. Silver nanoparticles were then deposited on BDD_L_ because of their selective electrochemical CO_2_ reduction ability. Excellent selectivity (estimated CO:H_2_ mass ratio of 318:1) and recyclability (stable for five cycles of 3 h each) for photoelectrochemical CO_2_ reduction were obtained for the optimum silver nanoparticle-modified BDD_L_ electrode at −1.1 V vs. RHE under 222-nm irradiation. The high efficiency and stability of this catalyst are ascribed to the *in situ* photoactivation of the BDD_L_ surface during the photoelectrochemical reaction. The present work reveals the potential of BDD_L_ as a high-energy electron source for use with co-catalysts in photochemical conversion.

Artificial photosynthesis is an emerging process for sustainable conversion of solar energy to chemical energy, and is important because of the increasing atmospheric CO_2_ concentration and global energy demand^1^,^2^,^3^. Artificial photosynthesis generally uses a semiconductor that absorbs solar radiation and generates electron/hole pairs^1^,^4^,^5^. These electron/hole pairs produce active radical species on the semiconductor surface prior to the adsorption of water or reacting molecules, or electron/hole pairs directly transfer to guest molecules through space^6^,^7^. The high energy of these active radicals and electron/hole pairs means they readily take part in certain chemical conversions under suitable conditions. For example, TiO_2_ is a well-known photocatalyst for water splitting and pollutant degradation^6^,^7^. TiO_2_ splits water into hydrogen and/or oxygen in the presence of a Pt counter electrode or hole scavenger using a photon energy greater than its bandgap. Other chemical conversions have also been achieved through semiconductor/molecular photocatalysis^8^,^9^,^10^,^11^. However, in multistep chemical conversion, which involve many intermediates to obtain a desired product, each intermediate has the possibility to divert the reaction course, resulting in poor product selectivity^12^,^13^,^14^. Although product selectivity in multistep reactions can be increased by choosing a suitable photocatalyst or controlling the reaction conditions, the reliability and efficiency of such reactions are still far from those required for commercial use^13^. Therefore, efficient conversion through multistep or multi-intermediate photochemical reaction remains challenging. Chemists typically attempt to divert such conversions into new mechanistic pathways with the fewest possible intermediates.

Photocatalytic or photoelectrochemical CO_2_ reduction in aqueous solution is one example where product selectivity is limited by both thermodynamics and kinetics. Commercial conversion of CO_2_ into high-value chemicals is generally inefficient, laborious, and expensive^12^,^13^,^14^. Efficient photocatalytic or photoelectrochemical CO_2_ reduction with excellent product selectivity is a research goal in the field of sustainable energy conversion, and represents an alternative to natural photosynthesis.

The atmospheric concentration of CO_2_ has increased tremendously over the last 50 years. Selective, efficient conversion of CO_2_ into a valuable chemical fuel is not only important for sustainability but also economically favourable^15^,^16^,^17^. Natural photosynthesis is an indispensable tool to capture CO_2_, but gross destruction of green plants and rapid civilization have forced researchers to search for alternatives to natural photosynthesis to efficiently and selectively convert CO_2_ into high-value chemicals. The selectivity of chlorophyll in natural photosynthesis is unprecedented. In artificial photocatalytic or photoelectrochemical CO_2_ reduction, conversion of CO_2_ into useful chemicals requires suitable catalysts^2^,^4^. Photocatalytic or photoelectrochemical CO_2_ reduction generally involves multiple proton-coupled electron transfer reactions in aqueous media. These reactions provide numerous intermediates, and thereby produce multiple CO_2_ reduction products^4^. Electrochemical or photoelectrochemical CO_2_ reduction has been explored using various catalysts with the aim of identifying mechanistic pathways that finally could lead to efficient conversion of CO_2_ into a particular product^12^,^18^,^19^,^20^,^21^,^22^.

Halmann *et al*.^22^. first reported photoelectrochemical CO_2_ reduction to produce formic acid, methanol and formaldehyde using a p-type GaP semiconductor in 1978. Since then, many semiconductor photocatalysts have been used for CO_2_ reduction^23^,^24^,^25^,^26^. Metal nanoparticles deposition on the semiconductors and fabrication of metal-semiconductor junction have successfully studied for improvement of photocatalytic CO_2_ reduction activity^27^,^28^,^29^. Recently, Hamers and co-workers found that hydrogen-terminated boron-doped diamond (H-BDD) can produce solvated electrons through injection of photoexcited electrons into aqueous electrolytes^30^. The solvated electrons produced by H-BDD can reduce CO_2_ into carbon monoxide (CO) alone via a one-electron transfer reduction mechanism under sufficiently high pressure. However, the extremely high pressure used in this approach is undesirable. In addition, H-BDD is easily oxidized under illumination^30^,^31^.

In this work, we study an alternative solution to overcome the challenges of one-electron transfer under normal pressure and temperature by using oxygen-terminated lightly boron-doped (1,000 ppm) diamond (BDD_L_) incorporated with co-catalysts. The surface of oxygen-terminated BDD_L_ is reduced during photoelectrochemical reaction so it acts as a semiconducting high-energy electron source^30^,^31^. Silver (Ag) co-catalyst deposited on BDD_L_ stabilizes the intermediates of CO_2_ reduction reactions under normal pressure to realize selective photoelectrochemical reduction. The semiconducting nature of BDD_L_ is investigated by changing the light sources used in photoelectrochemical studies.

## Results

### Surface morphology and photoelectrochemical study

**Figure 1:**
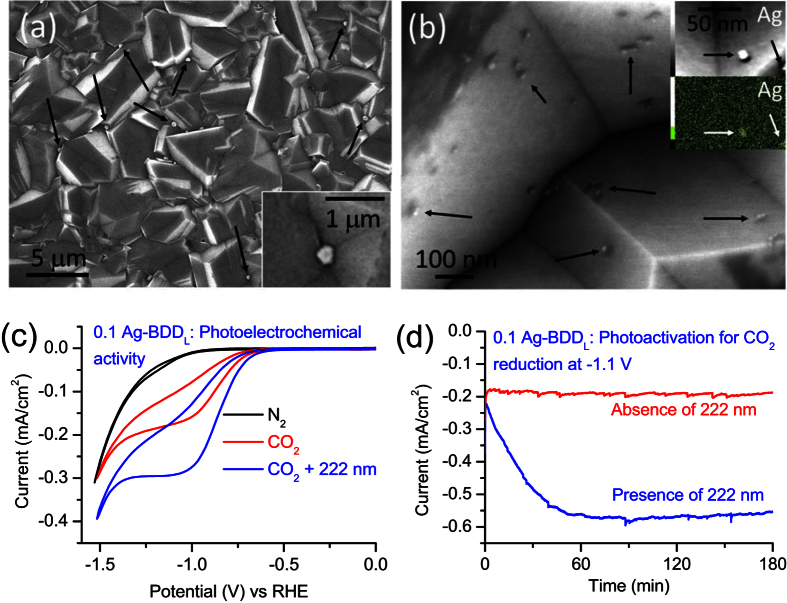
Surface morphology and photoelectrochemical CO_2_ reduction activity of Ag-BDD. (**a**) Low-magnification FESEM image of 0.1 Ag-BDD_L_ (0.1 Ag indicates that the AgNO_3_ concentration during deposition was 0.1 M; other parameters including potential and time were fixed at −0.5 V and 60 s, respectively), clearly indicating its smooth surface. Brighter Ag nanoparticles with a diameter of ~300 nm are indicated by arrows. (**b**) High-magnification FESEM image showing smaller Ag nanoparticles (~20 nm) as confirmed by elemental mapping (inset). (**c**) CVs of 0.1 Ag-BDD_L_ in 25 mM Na_2_SO_4_ aqueous electrolyte. The cathodic peak current at −1.1 V vs. RHE under CO_2_-saturated conditions indicates the cathodic reduction of CO_2_ on the 0.1 Ag-BDD_L_ electrode. (**d**) Chronoamperometric current–time curves of the photocurrent generated by the 0.1 Ag-BDD_L_ electrode in the dark and under irradiation (222 nm). Photocurrent increased in the first hour and then became almost constant.

Field-emission scanning electron microscopy (FESEM) and elemental analysis were used to investigate the deposition of Ag nanoparticles on BDD_L_. First, Ag nanoparticles were deposited on BDD_L_ by a chronoamperometric method in 0.1 M AgNO_3_ at −0.5 V for 60 s to produce a sample denoted 0.1 Ag-BDD_L_. Low-magnification FESEM analysis of this sample revealed that Ag nanoparticles with a diameter of ~300 nm were deposited on BDD_L_ (Fig. 1a). However, a high-magnification FESEM image indicated that primarily smaller Ag nanoparticles (average size ~20 nm) were deposited on BDD_L_ (Fig. 1b). The size distributions of smaller Ag nanoparticles are shown in Supplementary Fig. S1 by analysing several high magnified FESEM images. Average size of the smaller Ag nanoparticles was found to be around ~20 nm (Supplementary Fig. S1) with the 0.1 Ag-BDD_L_. The 0.1 Ag-BDD_L_ sample was then used for electrochemical/photoelectrochemical CO_2_ reduction. Figure 1c displays cyclic voltammograms (CVs) of a 0.1 Ag-BDD_L_ electrode in 25 mM Na_2_SO_4_. In N_2_, no cathodic peak was obtained up to −1.0 V vs. RHE, suggesting no reduction of Ag-BDD_L_ or the electrolyte. The low cathodic current density (onset potential −1.0 V vs. RHE) may indicate a poor hydrogen evolution reaction^32^. Indeed, under CO_2_-saturated conditions, 0.1 Ag-BDD_L_ displayed a strong cathodic peak at −1.1 V vs. RHE in 25 mM Na_2_SO_4_, clearly indicating the cathodic electrochemical reduction of CO_2_. The cathodic current density gradually decreased with increasing cycle number (Supplementary Fig. S2a). Careful observation revealed that the difference of cathodic current density between two consecutive cycles decreased as cycling progressed. After several cycles, the cathodic current density remained almost constant at −1.1 V (Supplementary Fig. S2a). The opposite behaviour was observed under irradiation at 222 nm with an excimer lamp (7 W). Under irradiation, the cathodic current density increased slowly and reached its maximum value after several cycles (Supplementary Fig. S2b). The increase in cathodic current density is attributed to the photoexcitation and photoactivation of 0.1 Ag-BDD_L_ by high-energy photons at −1.1 V vs. RHE. Therefore, under irradiation and applied bias potential, charge transport and cathodic CO_2_ reduction are facilitated, increasing cathodic current density. The final cycles in both the dark and light-irradiation conditions show quite different cathodic current densities (Fig. 1c). This large difference clearly reveals the excellent photoelectrochemical activity of the 0.1 Ag-BDD_L_ electrode for CO_2_ reduction.

To further confirm the photoelectrochemical behaviour of the 0.1 Ag-BDD_L_ electrode, partial current densities were measured in both the dark and light in 25 mM Na_2_SO_4_ at −1.1 V vs. RHE. In the first few minutes in the dark, the partial current density changed sharply from −0.3 to −0.2 V and then remained constant (Fig. 1d). This clearly agrees with the CV characteristics of Ag-BDD_L_ under CO_2_-saturated conditions. However, under photoexcitation at 222 nm, the partial current density at −1.1 V began to increase over time (Fig. 1d). This increase in partial current density is caused by the photoexcitation and photoactivation of 0.1 Ag-BDD_L_ and the subsequent participation of photoexcited electrons in the cathodic CO_2_ reduction reaction on the Ag surface, as revealed by CV analysis.

### Product analysis

**Figure 2:**
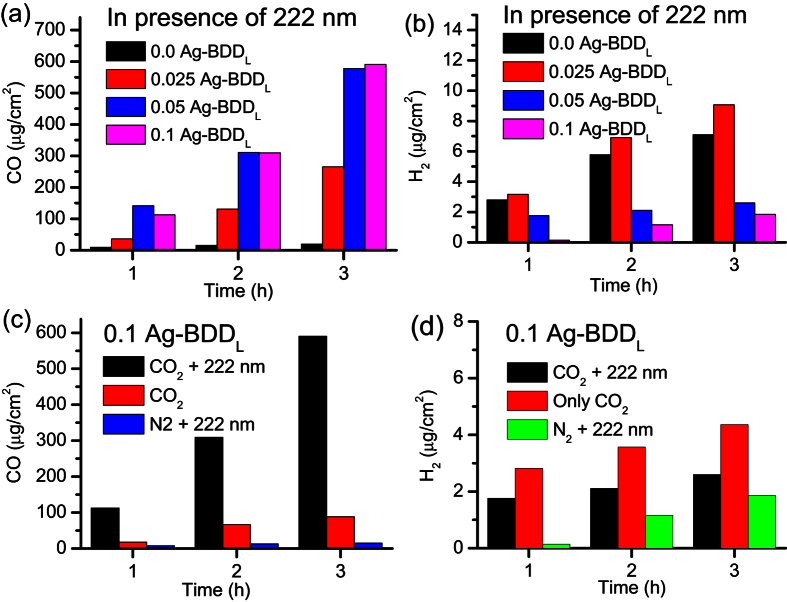
CO_2_ reduction product analysis. (**a**) Amount of CO produced during irradiation (222 nm) over different Ag-BDD_L_ electrodes in 25 mM Na_2_SO_4_ aqueous electrolyte at −1.1 V vs. RHE. At a particular duration, the amount of CO increases with the Ag concentration used during deposition on the BDD_L_ substrate. (**b**) Production of H_2_ over the electrodes. At a particular irradiation time, the amount of H_2_ decreased with increasing AgNO_3_ concentration during electrode synthesis. (**c**) Photoelectrochemical effect of 0.1 Ag-BDD_L_ under different conditions in 25 mM Na_2_SO_4_ at −1.1 V vs. RHE. The amount of CO produced under irradiation (222 nm) is higher than that produced in the dark, revealing that the very high amount of CO produced originates from the photoelectrochemical effect of 0.1 Ag-BDD_L_. Negligible CO was produced over 0.1 Ag-BDD_L_ under N_2_-saturated conditions. (**d**) Amount of H_2_ produced over the 0.1 Ag-BDD_L_ electrode under different conditions at −1.1 V, indicating that H_2_ is mostly produced through electrochemical reactions.

CV and chronoamperometric analysis clearly suggest the electrochemical and photoelectrochemical reduction of CO_2_ by the 0.1 Ag-BDD_L_ electrode at −1.1 V vs. RHE in 25 mM Na_2_SO_4_. Therefore, the CO_2_ reduction products obtained under similar conditions at −1.1 V vs. RHE with different amounts of Ag loaded on the BDD_L_ electrodes were detected. Gas chromatography (GC) and ion chromatography (IC) were used to investigate the reduction products. Figure 2 shows the amount of CO and H_2_ obtained using Ag-BDD_L_ electrodes with different Ag loading during irradiation (222 nm). The concentration of AgNO_3_ used during deposition was slowly increased from 0 to 0.1 M (0 indicates a bare BDD_L_ electrode, while 0.025 Ag-BDD_L_, 0.05 Ag-BDD_L_ and 0.1 Ag-BDD_L_ denote deposition of Ag from 0.025, 0.05 and 0.1 M AgNO_3_ aqueous solutions on BDD_L_ at −0.5 V for 60 s, respectively). The detailed of surface morphology with 0.025, and 0.05 Ag-BDD_L_ is shown in Supplement Fig. S1. Average sizes of the smaller Ag nanoparticles were found to be ~10 and ~15 nm with the samples 0.025, and 0.05 Ag-BDD_L_ electrodes, respectively. The photoelectrochemical CO_2_ reduction products obtained over these electrodes were analysed (Fig. 2a). The bare electrode (0 Ag-BDD_L_) produced a negligible amount of CO. The amount of CO produced increased markedly with Ag loading on BDD_L_. Although similar amounts of CO were obtained over 0.1 Ag-BDD_L_ (590 μg in 3 h) and 0.05 Ag-BDD_L_ (577 μg in 3 h), less H_2_ was formed by 0.1 Ag-BDD_L_ (1.85 μg in 3 h) than 0.05 Ag-BDD_L_ (2.59 μg in 3 h). Minor amount of H_2_ could be produced from competitive proton reduction in aqueous electrolyte. The weight ratios of CO to H_2_ were 29:1, 222:1 and 318:1 over 0.025 Ag-BDD_L_, 0.05 Ag-BDD_L_ and 0.1 Ag-BDD_L_, respectively. Therefore, the 0.1 Ag-BDD_L_ electrode produces CO with minimal H_2_ at −1.1 V vs. RHE under 222 nm irradiation.

The role of photoelectrochemical CO_2_ reduction at 0.1 Ag-BDD_L_ is emphasized by comparing the products obtained with and without light irradiation and under N_2_-saturated conditions at −1.1 V. GC analysis reveals that in the dark, the amount of CO produced (88 μg in 3 h) decreased considerably compared with that under irradiation, while the amount of H_2_ (4.35 μg in 3 h) remained similar (Fig. 2c,d). Similarly, under N_2_-saturated conditions, a negligible amount of CO (15 μg in 3 h) was detected, while the amount of H_2_ remained similar. Therefore, the greater amount of CO produced under CO_2_-saturated conditions and 222 nm irradiation results from the photoelectrochemical effect of 0.1 Ag-BDD_L_.

Ag-BDD_L_ could also produce liquid products because CO_2_ reduction often follows a multiple proton-coupled electron transfer reaction mechanism in aqueous electrolyte, providing numerous products. Therefore, IC was used to detect possible liquid products including formate, acetate and oxalate. No liquid products were detected from the Ag-BDD_L_ electrodes under CO_2_-saturated conditions in the light (222 nm) or dark at −1.1 V.

### Selectivity of different Ag-modified carbon electrodes

**Figure 3:**
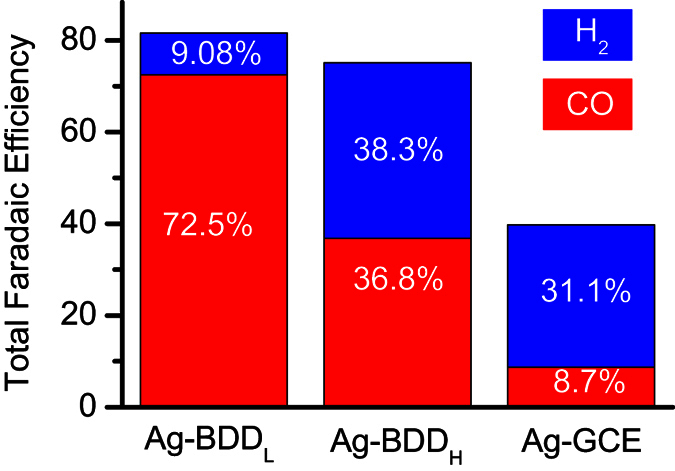
Selectivity of different carbon electrodes. Ag was deposited on BDD_L_, BDD_H_ and GCE in 0.1 M AgNO_3_ at −0.5 V for 60 s. Total Faradaic efficiency was measured after 3 h of photoelectrolysis at −1.1 V in 25 mM Na_2_SO_4_ under an excimer lamp (222 nm, 7 W).

We found that 0.1 Ag-BDD_L_ displayed more efficient and selective photoelectrochemical CO_2_ reduction than 0.05 and 0.025 Ag-BDD_L_ electrodes. To confirm the superior photoelectrochemical activity and selectivity of 0.1 Ag-BDD_L_, Ag nanoparticles were deposited on heavily boron-doped (10,000 ppm) diamond (denoted BDD_H_) and glassy carbon electrodes (GCEs) at −0.5 V for 60 s in 0.1 M AgNO_3_. The photoelectrochemical performance of the resulting electrodes was studied. Figure 3 shows the total Faradaic efficiency of these electrodes in 25 mM Na_2_SO_4_ at −1.1 V vs. RHE after 3 h of photoelectrolysis under 222 nm irradiation. The total Faradaic efficiency of 0.1 Ag-BDD_L_ was 81.5%. The Faradaic efficiency for CO with this electrode was 72.5%, and only 9.08% for H_2_, thereby providing very high selectivity for CO. Conversely, the total Faradaic efficiency of 0.1 Ag-BDD_H_ was 75.1%. Although this value is close to that of 0.1 Ag-BDD_L_, the Faradaic efficiency for CO of the 0.1 Ag-BDD_H_ electrode is only 36.8% and the CO:H ratio is ~1:1 indicating facile proton reduction at 0.1 Ag-BDD_H_. Meanwhile, 0.1 Ag-GCE exhibited a very poor total Faradaic efficiency (39.8%) with a Faradaic efficiency for CO of just 8.7%. Therefore, Ag-BDD_L_ displays increased conversion and selectivity in the photoelectrochemical reduction of CO_2_ in 25 mM Na_2_SO_4_ at −1.1 V vs. RHE compared with those of 0.1 Ag-BDD_H_ and 0.1 Ag-GCE.

### Stability and recyclability of the optimal photocathode

**Figure 4:**
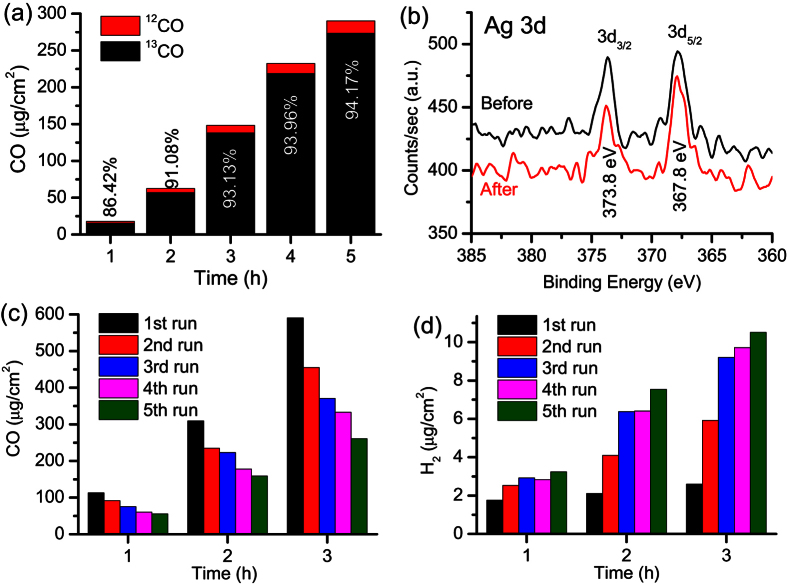
Stability and recyclability of the optimal photoelectrode. (**a**) Amount of isotopic ^13^CO and normal ^12^CO produced over time by the 0.1 Ag-BDD_L_ electrode in 25 mM Na_2_SO_4_ at −1.1 V vs. RHE. The amount of isotopic ^13^CO increased with irradiation time and reached 94.17% after 5 h. (**b**) XPS analysis of the 0.1 Ag-BDD_L_ electrode before and after photoelectrolysis at −1.1 V vs. RHE for 5 h in 25 mM Na_2_SO_4_ under 222 nm irradiation. The Ag 3d photoelectron peaks suggest the metallic state of Ag is not changed during the photoelectrochemical reaction. (**c**) Recyclability of the 0.1 Ag-BDD_L_ electrode in CO_2_-purged 25 mM Na_2_SO_4_ at −1.1 V. In each run, the electrolyte was purged with N_2_ and then CO_2_ for 1 h. The amount of CO produced decreased as the number of runs increased. (**d**) The amount of hydrogen produced in the consecutive runs indicates that hydrogen evolution increased with run number.

Electrode stability and recyclability are important factors that must be considered for their reliable use in photoelectrochemical solar energy conversion devices. Therefore, we studied the stability and recyclability of the optimized 0.1 Ag-BDD_L_ photocathode by performing an isotopic experiment because very high energy photons (222 nm, ~5 eV) could degrade the BDD_L_ surface or the resin used in the electrode contacts. The isotopic experiment was carried out by purging the 25 mM Na_2_SO_4_ electrolyte for 10 min with ^13^CO_2_ at a flow rate of 3 mL/min. Photoelectrochemical reduction was then carried out at −1.1 V vs. RHE under 222 nm irradiation and GC–mass spectrometry (GC-MS) was used to analyse the isotopic yield. Figure 4a shows the amounts of isotopic ^13^CO and normal ^12^CO after different periods. The amount of ^13^CO produced (267.56 μg after 5 h of illumination at −1.1 V vs. RHE) is lower than that of ^12^CO obtained previously (590 μg after 3 h; see Fig. 2c). This is because CO_2_ purging was carried out for 1 h in the previous experiment, so more CO_2_ was dissolved in the electrolyte compared with that in the case of isotopic ^13^CO_2_ (Fig. 1d, Supplementary Fig. S3). After illumination for 1 and 5 h, the amounts of isotopic ^13^CO were 86.42% and 94.17%, respectively. The increase in ^13^CO content is because only a little non-isotopic ^12^CO (16.8 μg after 5 h) could enter the system from the resin used to fabricate the electrode contact, which might be degraded by high-energy photons. The amount of ^12^CO produced by resin degradation is very small compared with that of ^13^CO produced through photoelectrochemical reduction. Therefore, the relative amount of isotopic ^13^CO increased over time. This isotopic experiment clearly reveals that CO originates from the reduction of CO_2_, not from degradation of resin, other organics or the BDD surface by the high-energy irradiation. Even after 5 h of 222 nm irradiation, 0.1 Ag-BDD_L_ was stable and produced ^13^CO with an excellent yield.

X-ray photoelectron spectroscopy (XPS) was performed before and after photoelectrocatalysis to investigate the elemental states of the 0.1 Ag-BDD_L_ electrode. Figure 4b displays the metallic Ag 3d photoelectron peaks^33^,^34^. The relative amount of Ag and nature of Ag 3d photoelectron peaks remained similar before and after photoelectrolysis at −1.1 V under 222 nm irradiation, indicating the oxidation states of the Ag nanoparticles did not change during photoelectrochemical operation; i.e., the metallic state of Ag is stable.

The recyclability of the 0.1 Ag-BDD_L_ electrode was examined by performing five runs for 3 h at −1.1 V in 25 mM Na_2_SO_4_. After each run, the cathodic peak position shifted to lower overpotential with slightly increased hydrogen evolution current compared with that at higher overpotential. After five runs, the cathodic peak for CO_2_ reduction moved to lower overpotential by 0.15 V with a current density of 0.2 mA/cm^2^ (Supplementary Fig. S4). The amounts of CO and H_2_ produced were measured (Fig. 4c,d). After five runs, the amount of CO produced in 3 h was 260 μg (44%), comparatively lower than that obtained for the first cycle (590 μg in 3 h). Conversely, the amount of H_2_ produced in the fifth cycle (10.51 μg) was greater than that obtained in the first (1.85 μg). The decrease in CO and increase in H_2_ produced with cycle number are attributed to the shift of the onset potential of CO_2_ reduction to lower overpotential as photoelectrolysis was conducted at −1.1 V (Supplementary Fig. S4).

## Discussion

Efficient photoelectrochemical CO_2_ reduction is very important for use of CO_2_ as a chemical feed stock and from an environmental viewpoint. Diamond is a highly stable material with a very wide bandgap (5 eV), possessing a wide electrochemical potential window, mechanical stability and biocompatibility^19^,^30^,^35^. Another advantage of diamond is that its conduction band (CB) position is very high, and hydrogen termination can shift its CB above the vacuum level, providing negative electron affinity towards photoexcited electrons^31^. Despite the very high energy of the photoexcited electrons of diamond, it is difficult to use under normal conditions. This is because pure diamond has very poor active sites for adsorption of foreign molecules. At sufficiently high pressure, the photoexcited electrons of hydrogen-terminated diamond or H-BDD can be used for photoelectrochemical conversion^30^,^31^. The photoexcited electrons in hydrogen-terminated diamond or H-BDD are capable of reducing CO_2_ via a one-electron transfer mechanism in 0.1 M Na_2_SO_4_ at sufficiently high pressure (2.5 MPa)^30^. Both the requirements of high pressure and hydrogen termination of the catalyst limit the application of diamond as a catalyst. Therefore, here we modified oxygen-terminated BDD_L_ with Ag nanoparticles to realize photoelectrochemical CO_2_ reduction under normal pressure and temperature. Ag nanoparticles were deposited on BDD_L_ because Ag is known for its selective electrochemical CO_2_ reduction^36^,^37^. The selectivity of Ag originates from its ability to form strong chemical bonds with CO_2_ under a certain applied potential, which is very important for product selectivity^36^,^37^,^38^. Strong chemical bonding of a catalyst surface with CO_2_ reduction intermediates can terminate the reduction reaction at a particular point without further propagation, unlike Cu surfaces, which are known to further propagate reduction reactions to produce multiple CO_2_ reduction products^12^,^36^.

Photoelectrochemical studies and product analysis revealed that the optimized 0.1 Ag-BDD_L_ electrode exhibited high efficiency and selectivity in the formation of CO in aqueous electrolyte at −1.1 V vs. RHE with a Faradaic efficiency of 72.5% after 3 h under 222 nm irradiation (Fig. 3). Photoelectrochemical studies using this optimized 0.1 Ag-BDD_L_ electrode were also carried out under similar conditions with 172- and 308-nm irradiation. Irradiation at 308 nm induced a small increase of CO production (235 μg in 3 h). Conversely, 172-nm irradiation yielded less CO (97 μg in 3 h), similar to -electrochemical CO production alone over the same electrode (Fig. 2c). The marked photoelectrochemical effect of 222 nm irradiation on the performance of the optimized 0.1 Ag-BDD_L_ electrode reveals the role of excitation of valence band (VB) electrons to the CB (Figs 1c,d and 2, Supplementary Fig. S5) in CO production. This is because the band gap of BDD_L_ (~5 eV) matches well with a 222 nm light source.

Bare BDD_L_ irradiated with 222 nm light did not efficiently produce CO_2_ reduction products (~19 μg CO that originated from the degradation of impurities; see Fig. 4a) under similar experimental conditions, as shown in Fig. 2a and Supplementary Fig. S6. This is consistent with the finding of Hamers *et al*.^30^. that photoexcited electrons produced in a BDD semiconductor could not reduce CO_2_ into CO under normal pressure (Supplementary Fig. S6). Thus, the electrochemical CO_2_ reduction activity of Ag-BDD_L_ is related to the presence of Ag nanoparticles^36^. However, electrochemical reduction of CO_2_ by Ag-BDD_L_ in aqueous electrolyte at −1.1 V vs. RHE decreased markedly after a few minutes and then became poor (Fig. 1d). This behaviour is also evident from the consecutive CV runs conducted under CO_2_-saturated conditions in the dark (Supplementary Fig. S2a). In the dark, the cathodic peak current obtained at −1.1 V vs. RHE in 25 mM Na_2_SO_4_ decreased until it reached a constant value (Supplementary Fig. S2a). Conversely, the cathodic peak current increased until it reached its maximum value after a certain irradiation time under 222 nm irradiation at −1.1 V vs. RHE. This increase in photocurrent (Fig. 1d, Supplementary Fig. S3) is thought to be caused by two factors: first, photoreduction of the BDD_L_ surface produces photoactive sites at high negative applied potential, and second, simultaneous enhanced photoelectrochemical reduction of CO_2_ on the Ag surface. This situation was confirmed by XPS analysis. The C 1 s peaks of 0.1 Ag-BDD_L_ before and after photoelectrolysis at −1.1 V vs. RHE in 25 mM Na_2_SO_4_ for 5 h are presented in Supplementary Figs S7 and S8. A broader C 1 s peak [full width at half maximum (FWHM) of 2.73 eV] is obtained before photoelectrochemical reaction, and becomes narrower (FWHM=1.51 eV) after photoelectrolysis for 5 h at −1.1 V in the presence of 222 nm irradiation (Supplementary Fig. S8). This is because BDD_L_ is oxygen terminated, so a small amount of C-O-C and C=O/C-OH bonds are present in addition to C-C bonds at its surface. The C=O/C-OH bonds might be reduced at −1.1 V under 222 nm irradiation. Therefore, the C 1 s peak displays greater C-C sp^3^ character after photoelectrolysis because of the decreased amount of C=O/C-OH bonds, causing it to narrow (Supplementary Fig. S8). Note that electrochemical bias potential alone cannot reduce the diamond surface; 222 nm irradiation is needed for BDD surface reduction, as evident from XPS, CV, and chronoamperometric analyses (Fig. 1c,d; Supplementary Figs S7 and S8). Our results reveal that 0.1 Ag-BDD_L_ is activated under 222 nm irradiation at −1.1 V vs. RHE to provide very high photoelectrochemical CO_2_ reduction activity.

There are three plausible formation pathways of CO from CO_2_ electrochemical or photoelectrochemical reduction:^30^,^38^,^39^


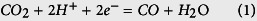



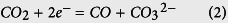



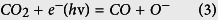


**Figure 5:**
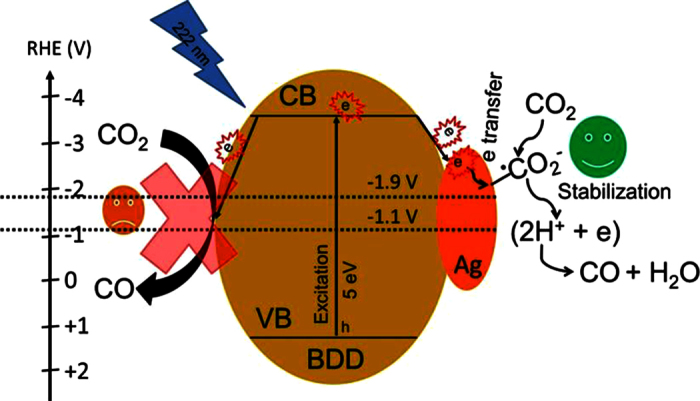
Schematic diagram of half-cell charge transfer with an Ag-BDD_L_ photocathode in 25 mM Na_2_SO_4_ for CO_2_ reduction. Photoexcited electrons are produced in BDD_L_ under 222 nm irradiation. The photoexcited electrons are easily transferred to the Ag nanoparticles deposited on BDD_L_. These photoexcited electrons could be transferred to the CO_2_ molecules adsorbed on the Ag surface to form 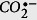 anion radicals. The highly energetic 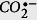 anion radicals are readily stabilized by the proton-coupled electron transfer mechanism to produce CO at −1.1 V vs. RHE. For clarity, donor levels near the VB of BDD_L_ originating from the boron impurities are not shown.

In aqueous electrolyte, CO is mostly formed from CO_2_ via reaction (1), where 2 H^+^ and 2*e*^−^ participate. In organic solvent/aprotic electrolyte, reaction (2) is favoured at sufficiently high overpotential or high pressure^39^,^40^. Generally, reaction (3) is rare for photoelectrochemical CO_2_ reduction because reduction is usually carried out at negative bias potential, so such photodissociation on the electrode surface is difficult^30^. Note that in aqueous media, hydrogen is often produced through proton reduction^41^, e.g., 2*H*^+^ + 2*H*^+^ + 2*e*^−^ = *H*_2_2*e*^−^ in addition to CO_2_ reduction at the electrode surface. A negligible amount of hydrogen found with the Ag-BDD_L_ electrode could produce via proton reduction. However, the experimental results reveal that this smaller amount of hydrogen is produced through electrochemical reduction of proton at the BDD surface (Figs 2 and 3). Hori *et al*.^36^. reported that Ag, Au and Zn can electrochemically reduce CO_2_ to CO with high selectivity. This is possibly because of the formation of strong chemical bonds between 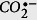 and the catalyst surface. Formation of the 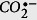 anion radical involves one-electron transfer (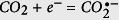), and this anion species is very unstable. The high photoelectrochemical activity of 0.1 Ag-BDD_L_ in aqueous electrolyte at −1.1 V vs. RHE is attributed to the transfer of very high energy photoexcited electrons in the CB of BDD_L_ to the Ag nanoparticle surfaces to facilitate CO_2_ reduction. The electrochemical cathodic peak of the 0.1 Ag-BDD_L_ electrode under CO_2_-saturated conditions suggests that CO_2_ reduction occurs on the Ag surface. Therefore, the intermediates of CO_2_ reduction must be adsorbed on the Ag surface^37^. It has been reported that Ag surfaces could stabilize 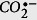 anion radicals at certain negative potential depending on the experimental conditions, electrolyte and pH (standard redox potential for 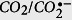 is −1.9 V)^12^,^36^,^37^,^42^. BDD can produce the 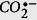
anion radical in 0.1 M Na_2_SO_4_ and this radical can be stabilized as an intermediate product under suitable experimental conditions (high pressure)^30^. Therefore, the enhanced photoelectrochemical CO_2_ reduction of 0.1 Ag-BDD_L_ is thought to be related to the formation of 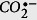 anion radicals by the transfer of photoexcited electrons to the Ag nanoparticles. The produced 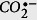 anion radicals are then immediately stabilized by a proton-coupled electron transfer mechanism at the Ag surface (Fig. 5) to produce the more stable products CO and H_2_O^37^.

## Conclusion

In summary, the optimized Ag-BDD_L_ electrode exhibits enhanced photoelectrochemical CO_2_ reduction activity for CO formation with minimal H_2_ production under 222 nm irradiation. This Ag-BDD_L_ electrode exhibits superior activity to that of highly doped BDD_H_ and GCE modified with Ag nanoparticles. The optimized Ag-BDD_L_ electrode is stable after five cycles, retaining 44% of its initial activity for CO production. A high isotopic yield (94.17% after 5 h) revealed that the electrode is not corroded under 222 nm irradiation at −1.1 V. The content of surface oxygenated species decreases slightly during the photoelectrochemical reaction, which might increase the number of active sites for CO_2_ reduction. Therefore, the photocurrent increases during the first hour of photoelectrochemical reaction with the 0.1 Ag-BDD_L_ electrode. Such surface photoreduction was not observed after 1 h of photoelectrochemical reaction. The enhanced activity of Ag-BDD_L_ is ascribed to the synergistic interaction of the Ag nanoparticles with semiconducting BDD_L_. Ag is known for its selective CO_2_ reduction, while the very high energy of the CB of BDD_L_ enhanced the formation of 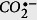 anion radicals on the Ag surface in aqueous electrolyte. The 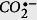 anion radicals were stabilized on the Ag surface at −1.1 V via a proton-coupled electron transfer mechanism to produce CO. The very high energy of the CB electrons of BDD_L_ could be coupled with other co-catalysts to potentially improve the yield of several kinetically and thermodynamically limited photochemical conversions.

## Methods

### Deposition of Ag nanoparticles

BDD thin films were fabricated on silicon substrates using a reported microwave plasma chemical vapour deposition method^35^. Ag nanoparticles were electrodeposited on BDD_L_ (effective area 1 × 1 cm) in a two-electrode system at −0.5 V with respect to a Pt counter electrode in aqueous AgNO_3_ (0.025–0.1 M) for 60 s. After Ag nanoparticle deposition, the electrodes were cleaned by sonication in deionized water (18.2 MΩ) for 15 min. After washing, electrodes were dried under a N_2_ flow at room temperature.

### Photoelectrochemical studies

Photoelectrochemical CO_2_ reduction activity was investigated in an H-type electrochemical cell. Working and counter electrodes were separated by a proton-exchange Nafion membrane, and an aqueous solution of Na_2_SO_4_ was used as the supporting electrolyte. Ag/AgCl saturated with KCl was used as a reference electrode and Pt mesh served as a counter electrode. The scan rate was 100 mV/s. Excimer lamps (7 W) with wavelengths of 172, 222 and 308 nm were used in the photoelectrochemical studies. Light sources were positioned 2 cm away from the electrode and the reaction temperature was held at 25 °C throughout the experiments.

### Gas chromatography analysis

Gaseous products were analysed by a gas chromatograph (GC-2014, Shimadzu, Japan) equipped with a thermal conductivity detector and flame ionization detector. Isotopic ^13^CO was measured by GC-MS (GC-2010 Plus, Shimadzu) and the relative amount was estimated by comparison with the amount of ^12^CO produced.

### Ion chromatography analysis

An IC system (Dionex ICS-1000, Thermo Scientific) equipped with DionexIonPac AS12A and DionexIonPac CS12A columns was used to identify any detectable CO_2_ reduction liquid products obtained with the Ag-modified BDD_L_ electrodes.

### Sample characterization

An FESEM (JEOL, JEM-3100F) was used to analyse the surface morphology of the Ag-BDD_L_ electrodes. Energy-dispersive X-ray spectroscopy was conducted for elemental mapping of the Ag nanoparticle-modified BDD_L_ substrates. Elemental states of Ag-BDD_L_ electrodes before and after photoelectrochemical reaction at −1.1 V for 5 h were determined with an X-ray photoelectron spectrometer (ESCA-3400, Shimadzu) using a monochromatic Mg Kα source.

## Additional Information

**How to cite this article**: Roy, N. *et al*. Boron-doped diamond semiconductor electrodes: Efficient photoelectrochemical CO_2_ reduction through surface modification. *Sci. Rep.* **6**, 38010; doi: 10.1038/srep38010 (2016).

**Publisher's note:** Springer Nature remains neutral with regard to jurisdictional claims in published maps and institutional affiliations.

## Supplementary Material

Supplementary Information
