# Supplementary material for: Boron-doped diamond semiconductor electrodes: Efficient photoelectrochemical CO2 reduction through surface modification
Source: Sci Rep. 2016 Nov 28;6:38010. doi: 10.1038/srep38010 (PMC5125091; doi:10.1038/srep38010)
Supplement: Supplementary Information [file srep38010-s1.pdf]

Supplementary Information

Boron-doped diamond semiconductor electrodes: Efficient photoelectrochemical CO_2 reduction through surface modification

Nitish Roy[1], Yuiri Hirano[1,2] , Haruo Kuriyama[3], Pichaimuthu Sudhagar[4], Norihiro Suzuki[1], Ken-ichi Katsumata[1], Kazuya Nakata[1,2], Takeshi Kondo[1,2], Makoto Yuasa[1,2], Izumi Serizawa[3], Tomoaki Takayama[5], Akihiko Kudo[1,5], Akira Fujishima[1,*], & Chiaki Terashima[1,*]

[1]*Photocatalysis International Research Center, Tokyo University of Science, 2641 Yamazaki, Noda, Chiba 278-8510, Japan*

[2]*Faculty of Science and Technology, Tokyo University of Science, 2641 Yamazaki, Noda, Chiba 278-8510, Japan*

[3]*ORC Manufacturing Co., Ltd, 4896 Tamagawa, Chino, Nagano 391-0011, Japan*

[4]*Environmental and Sustainability Institute, University of Exeter, Penryn, Cornwall TR10 9EZ, UK*

[5]*Faculty of Science, Tokyo University of Science, 1-3 Kagurazaka, Shinjuku, Tokyo 162-8601, Japan*

(e-mail: terashima@rs.tus.ac.jp or A. F. fujishima_akira@admin.tus.ac.jp)

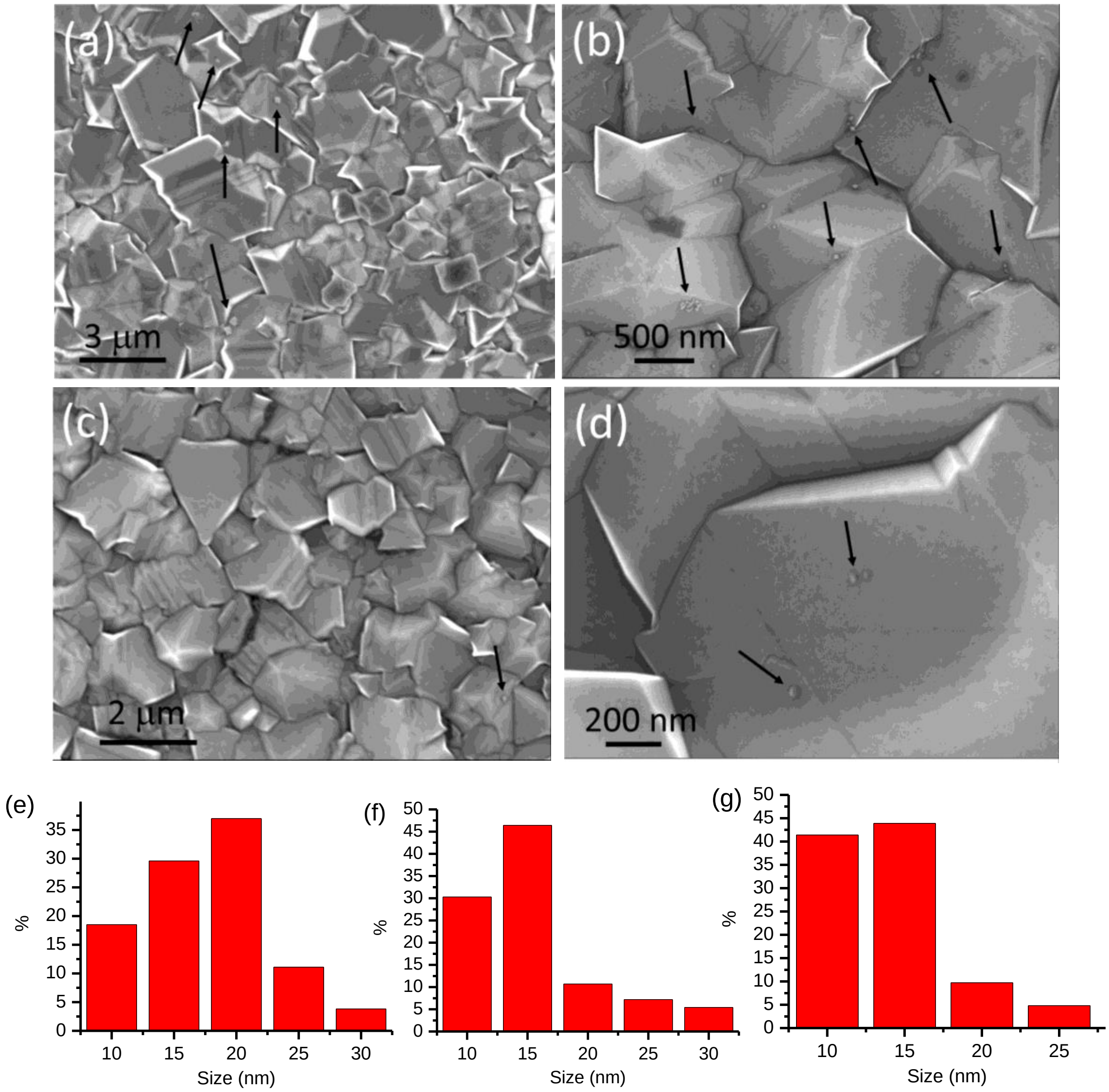

Supplementary Figure S1. (a, b) Low- and high-magnification FESEM images of 0.05 Ag-BDD_L, and **(c, d)** 0.025 Ag-BDD_L. Arrows indicate the deposition of Ag nanoparticles on BDD_L by chronoamperometry at −0.5 V in aqueous $AgNO_3$ solution. **(e, f, g)** Size distributions of the smaller Ag nanoparticles on BDD_L with 0.1, 0.05, and 0.025 Ag-BDD_L, respectively. Distribution plots were calculated based on several high magnification FESEM images.

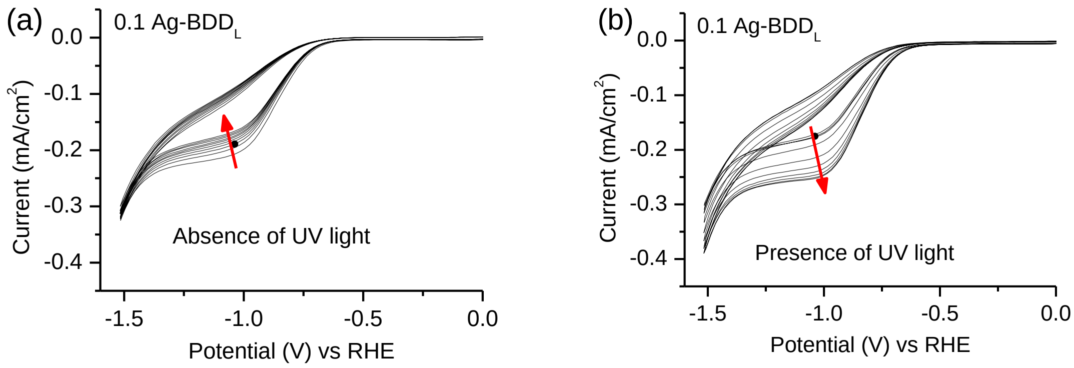

Supplementary Figure S2. (a) Consecutive cyclic voltammograms (CVs) of 0.1 Ag-BDD$_L$ in 25 mM Na_2SO_4 in the dark. The arrow indicates the decrease in peak current density showing a decrease in activity over time for electrochemical reduction of CO_2 over the 0.1 Ag-BDD$_L$ electrode. **(b)** Consecutive CVs of 0.1 Ag-BDD$_L$ in the same electrolyte under 222-nm irradiation indicating photoactivation and enhanced photoelectrochemical reduction (the arrow shows the increase in peak current) of CO_2 at −1.1 V vs. RHE compared with that in the dark.

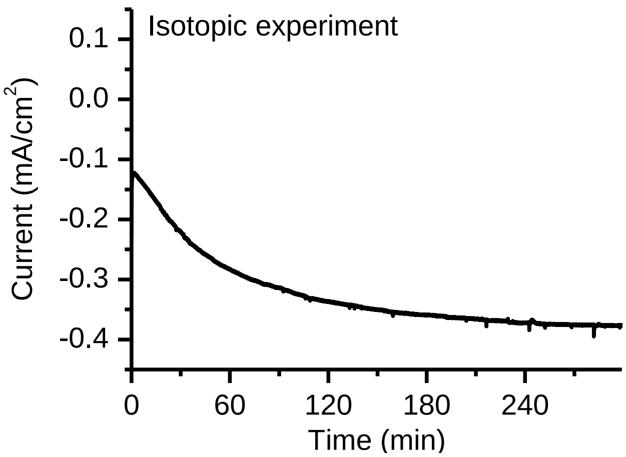

Supplementary Figure S3. Isotopic current–time curve at −1.1 V vs. RHE after purging the 25 mM Na_2SO_4 aqueous electrolyte with 30 mL of $^{13}CO_2$.

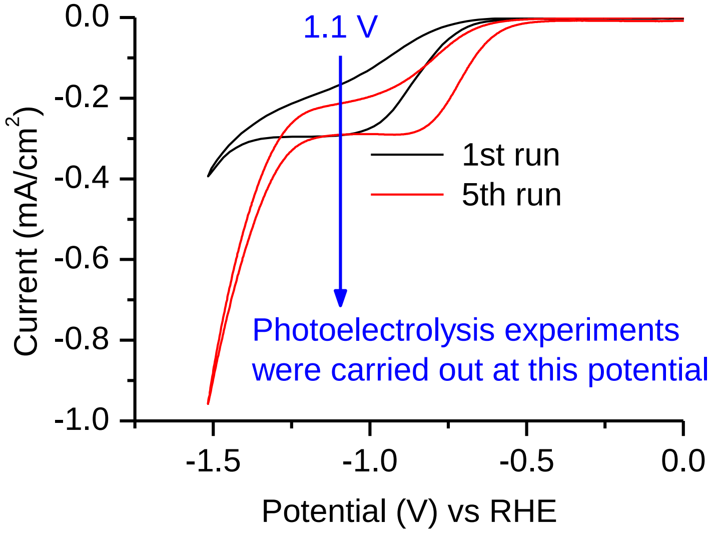

Supplementary Figure S4. CVs collected before the first and fifth photoelectrochemical runs for 0.1 Ag-BDD$_L$ under CO_2-saturated conditions and 222-nm irradiation in 25 mM Na_2SO_4aqueous solution.

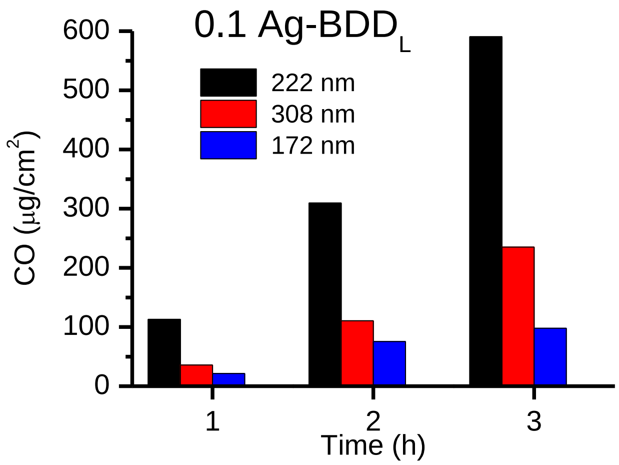

Supplementary Figure S5. Amount of CO produced by the 0.1 Ag-BDD$_L$ electrode under different light sources in 25 mM Na_2SO_4 aqueous electrolyte at −1.1 V vs. RHE, indicating the superior photoexcitation from the VB to CB of BDD$_L$ by a 222-nm light source compared with that of sources of other wavelengths. This is because the bandgap of BDD$_L$ is ~5 eV, so the 222-nm light source excites the VB electrons more efficiently than the other ones. The small enhancement of CO production under 308-nm irradiation is caused by the small amount of defects in BDD$_L$ (origination from boron doping and oxygen termination), and thereby a small amount of photoexcitation of BDD$_L$ occurs under the 308-nm light source. Conversely, 172-nm irradiation produces a similar amount of CO to electrochemical CO production alone at −1.1 V, indicating the poor photodissociation of CO_2 to form CO.

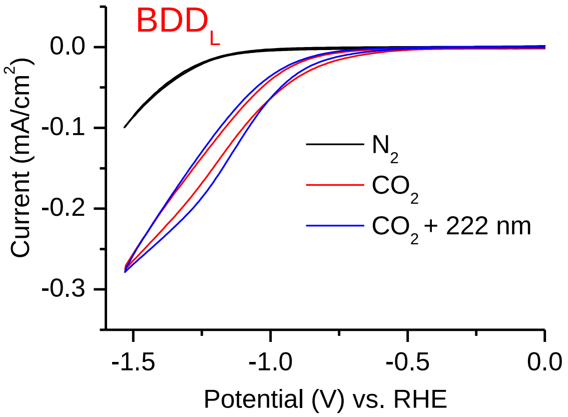

Supplementary Figure S6. CVs of bare BDD$_L$ (0.0 Ag-BDD$_L$) in 25 mM Na_2SO_4 aqueous electrolyte under different conditions.

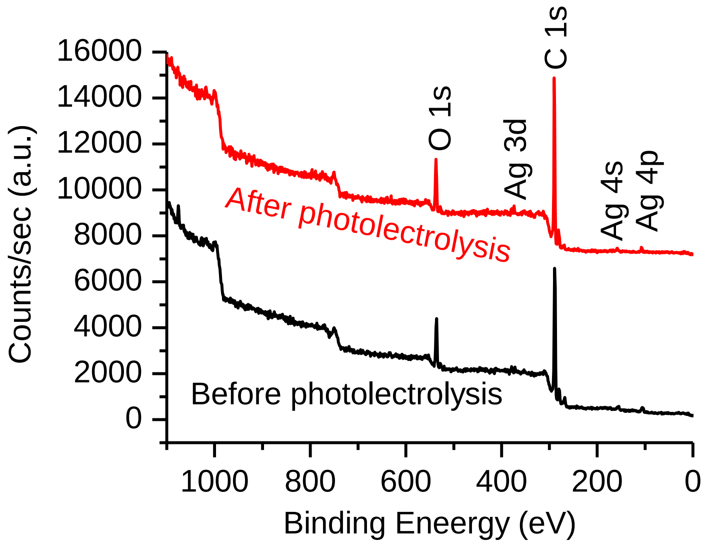

Supplementary Figure S7. X-ray photoelectron surface survey spectra of 0.1 Ag-BDD_L electrode before and after photoelectrochemical reaction for 5 h in 25 mM Na_2SO_4 under CO_2-saturated conditions at a bias potential of −1.1 V.

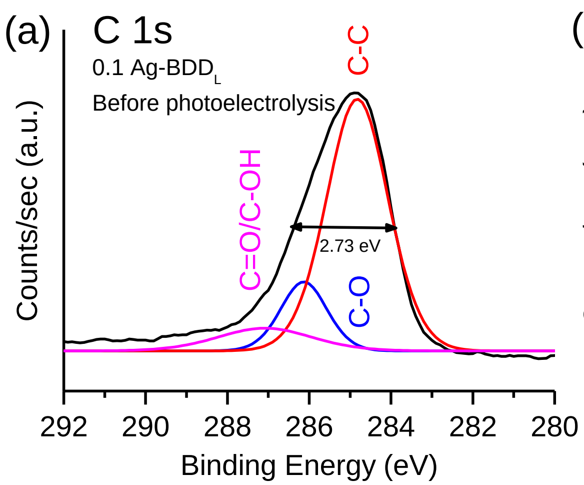

Supplementary Figure S8. (a) XPS C 1s peaks of 0.1 Ag-BDD$_L$ before photoelectrochemical reaction. The peaks are the sum of C-C sp^3, C-O and C=O/C-OH, which are shown as different colours. (b) XPS C 1s peaks of 0.1 Ag-BDD$_L$ after photoelectrolysis at −1.1 V in 25 mM Na_2SO_4 aqueous electrolyte under CO_2-saturated conditions and 222-nm irradiation for 5 h. The peaks are the sum of C-C sp^3, C-O and C=O/C-OH, which are shown as different colours. After photoelectrolysis, the relative amount of C=O decreased considerably, while the C-O peak decreased slightly compared with the C-C sp^3 one.
